# Indiana jones and ganser syndrome

**DOI:** 10.1192/j.eurpsy.2021.1972

**Published:** 2021-08-13

**Authors:** M. Pérez Fominaya, M.V. López, A. Osca

**Affiliations:** Psiquiatria, Hospital Universitario Nuestra Señora del Prado, Toledo, Spain

**Keywords:** ganser, hysteria, old age

## Abstract

**Introduction:**

Ganser syndrome is a rare medical disorder situated between hysterical etiopathogenesis and psychosis, factitious disorders and organic lesions which results in a difficult diagnosis. It is listed in DSM VI as a dissociative disorder whose main symptom is approximate answers, other accompanying symptoms appearing in Ganser: clouding of consciousness, somatic conversion symptoms and hallucinations. Psychopathologically explains a mental state of escape from a situation difficult to being tolerated. Organically appears in pathologies involving the frontal lobes

**Objectives:**

We present a case of a XX year old man who suddenly develops a depressive disorder with no apparent cause. Initially he was a professionally developed man. Famous archaeologist. University professor. Guitarist in a musical group. He deteriorated through the years, appearing dementia data with auditive hallucinations and resistant headache. He begins to have difficulty speaking correctly, with paraphasias and short answers, continually repeating “I don´t know”, Short-term amnesia and bed-chair life

**Methods:**

It was impossible to perform both the Mini-Mental State Examination test and the fototest because the patient refused claiming to be very nervous. The CT and MRI showed a slight temporary atrophy and vascular age changes. Subsequently, PET was performed without notable findings. Antidepressant and anxiolytic treatment was introduced without success as well as treatment for dementia and antipsychotic treatment.

**Results:**

The headache was improved. The rest of the symptoms did not disappear

**Conclusions:**

Ganser syndrome is a psychiatric condition that is difficult to diagnose and treat.

**Disclosure:**

No significant relationships.

